# Registration of the Cone Beam CT and Blue-Ray Scanned Dental Model Based on the Improved ICP Algorithm

**DOI:** 10.1155/2014/348740

**Published:** 2014-01-05

**Authors:** Xue Mei, Zhenhua Li, Songsong Xu, Xiaoyan Guo

**Affiliations:** Automation & Electrical Engineering School, Nanjing University of Technology, Nanjing, Jiangsu 211816, China

## Abstract

Multimodality image registration and fusion has complementary significance for guiding dental implant surgery. As the needs of the different resolution image registration, we develop an improved Iterative Closest Point (ICP) algorithm that focuses on the registration of Cone Beam Computed Tomography (CT) image and high-resolution Blue-light scanner image. The proposed algorithm includes two major phases, coarse and precise registration. Firstly, for reducing the matching interference of human subjective factors, we extract feature points based on curvature characteristics and use the improved three point's translational transformation method to realize coarse registration. Then, the feature point set and reference point set, obtained by the initial registered transformation, are processed in the precise registration step. Even with the unsatisfactory initial values, this two steps registration method can guarantee the global convergence and the convergence precision. Experimental results demonstrate that the method has successfully realized the registration of the Cone Beam CT dental model and the blue-ray scanner model with higher accuracy. So the method could provide researching foundation for the relevant software development in terms of the registration of multi-modality medical data.

## 1. Introduction

With the rapid development of the image processing, reverse engineering, computer-aided design, and image guidance play an important role in dental implant surgery [1]. A number of dental and jaw imaging modalities, including video imaging [2], Computed Tomography (CT) [3], and magnetic radiotherapy imaging (MRI) [4], are used in image-aided dental implant surgery. Performing the multimodality image registration from different imaging devices has complementary significance for guiding dental implant surgery. When dentists design schemes, they want to consider the morphology of the soft tissue and the hard tissue together. CT image and visible spectrum image fusion models [5] can multiply various factors and help the dentists achieve the optimal implant scheme.

Traditional registrations of Cone Beam Computed Tomography (CBCT) and other three-dimensional scanner models mainly rely on the fiducial markers method, which need to manually select the feature points. In [6–8], manual feature selecting methods have been tried for the registration of the mandible CT and plaster cast of the dental models. Because the images are complex and the location accuracy is limited by the experience of the operator and the operating state, the manual feature selecting method might provide misleading information and lead to instability problems. Noh et al. [9] designed an occlusal form board, with titanium spheres markers, to match the image using the titanium spheres markers as feature points. In this model, the position of makers is stable, but the drawback is the extra burden for patients. The methods mentioned above cannot perfectly meet the needs of developing image-aided dental surgery. Some attempts have been made to select the feature point set automatically. Two main difficult problems are taken out in the process of the registration; one is the feature point selection and the other is the merge of different modality images with different resolutions.

Iterative Closest Point (ICP), in recent years, many researchers have extended or improved the traditional ICP algorithm. Xie et al. [10] proposed the fractional ICP (FICP) algorithm to trim abnormal points effectively, which enhanced the robustness of the ICP algorithm. Choi et al. [11] proposed the improved *k*-*d* trees traversal method to accelerate finding process of the nearest points. Bae and Lichti [12] further extended the improved ICP method based on the boundary feature points of the point cloud, which improved the efficiency and accuracy of traditional ICP algorithm greatly.

The aim of this work is to develop an improved Iterative Closest Point (ICP) algorithm in terms of the problem of registration with different image resolutions. Firstly, extract feature point set based on curvature characteristics, and then achieve the registration results by two procedures: the coarse registration and the precise registration. To verify the reliability of the improved ICP algorithm, we test it with 3D CBCT dental model and inEos Blue-light scanned model. These two models provide reconstructed CT dental data and visible light scanned dental-surface data, respectively. Experimental results show that the accuracy is increased significantly and this solution meets the requirements of the image-assisted surgery.

The rest of this paper is organized as follows.  Section 2 provides an overview of the ICP algorithm and proposes an improved ICP algorithm.  Section 3 describes the application of the improved ICP algorithm.  Section 4 reports the results of experiments conducted on the CBCT model and the inEos blue scanned model and provides the discussions. Conclusions are presented in Section 5.

## 2. The Improved ICP Algorithm

Generally, different medical imaging equipment differs in imaging mechanism, which contributes to different emphases on image information and makes image resolution difference. Thus, it results in complex difficulties in the image fusion; for example, the preprocessed 3D CBCT dental model contains two parts: dental crowns and roots, while the 3D inEos Blue-light dental model contains dental crowns only. Additionally, the resolution of the CBCT model is 0.625 mm, while that of inEos Blue-light scanner is 0.1 *μ*m. In view of data characteristics above, an improved ICP algorithm is proposed in this paper. First of all, feature point set of the three-dimensional surface images was extracted based on curvature characteristics to realize the coarse registration and to achieve the transformation matrix. Then, the feature point set and reference point set, obtained by the initial registered transformation, are processed in the precise registration step using ICP.

### 2.1. ICP Algorithm

ICP algorithm is commonly used as registration method, which enables the minimum mean square error in the distance of corresponding point or point surface between two point sets approaching the minimal by iterative calculations [13]. It repeats the process of calculating the optimal rigid transformation of the corresponding point set until convergence criterion is satisfied; that is, it has met the precision of registration. The transformation relation is shown as function (1), and the convergence criterion is shown as function (2):

(1) $$\begin{array}{c} Q_{i}={RP}_{i}+T, \end{array}$$

(2) $$\begin{array}{c} e=\sum_{i=1}^{N}{||Q_{i}-\left({RP}_{i}+T\right)||}^{2}, \end{array}$$

where *P*_*i*_ and *Q*_*i*_ present point sets corresponding to two images, respectively, *R* is a rotation matrix, *T* is a translation vector, and *e* is the error. When the error reaches the minimum, the convergence criterion meets requirement [14].

The traditional ICP algorithm used in registration requires one of the two point sets to be the subset of the other one. The relatively initial positions of two point sets are limited strictly in ICP, which ensure no big gap between the two point sets. When the constraints are unsatisfied or the huge deviation exists, it will affect the processing results and make the registration unreliable.

### 2.2. The Selection of Feature Point Sets

The data of the CBCT dental model and the inEos Blue scanned model are both stored in STL file form and 3D models. The image surface discrete curvatures are sampled as the feature set. Because the STL Model is comprised of many triangular meshes, an improved Voronoi method is implemented to calculate vertex curvature [15].

**Figure 1:**
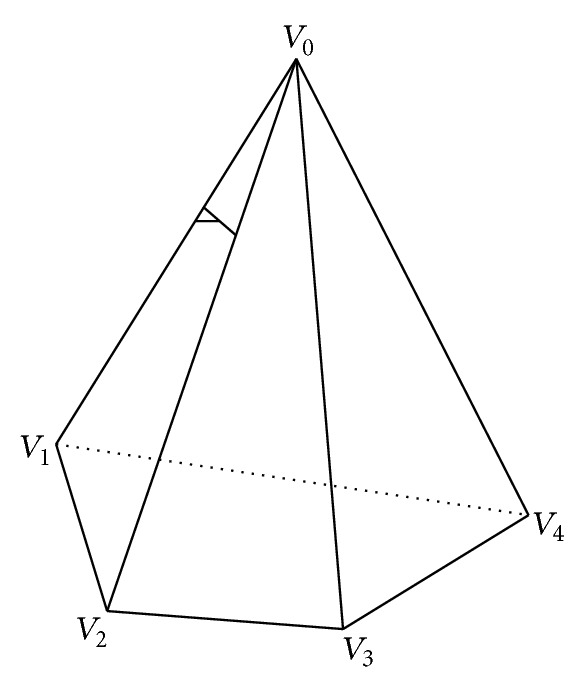
Triangular mesh in STL file.

As shown in Figure 1, *V*_0_ is a vertex of the network model. The curvature of *V*_0_ can be calculated by

(3) $$\begin{array}{c} K=2\pi -\frac{\sum_{j}\theta_{j}}{A}\;\left(j=1,2,\ldots ,N\right), \end{array}$$

where *θ*_*j*_ is the angle between the side *V*_0_ *V*_*j*_ and the side *V*_0_ *V*_*j*+1_, and *A* is the area of all triangle meshes which are connected with *V*_0_.

Set *α* as the threshold. When the curvature of one point is greater than *α*, it will be marked as feature point and put it into alternative feature list. The feature point set is defined as *P* = {*p*_*i*_ | *p*_*i*_ ∈ *R* ^3^; *i* = 1,2, 3,…, *N*_*P*_}.

### 2.3. The Coarse Registration

The improved three point's translational transformation method is used in this paper based on curvature characteristics to calculate rotation matrix *R* and translation vector *T*, so as to realize the semiautomatic extraction of corresponding points for two dental image models. At the same time, this method can reduce the interference of human factors in the matching process and enhance the accuracy of coarse registration.

#### 2.3.1. The Improved Three Point's Translational Transformation Method

For the two image models, one is defined as reference model and the other is defined as target model. Select three points, whose curvatures in the top 10 percent and distributions are homogeneous, as the reference points from the reference model and the corresponding points are in target model. After achieving reference points, we calculate the curvature of each vertex in target model and compare it with their value of the reference points. When the error is smaller than the setting threshold, mark this point as the centralized point of corresponding points and put it into corresponding points list. Due to the fact that there are some feature points with the same or similar curvature, it is common to lead to one reference point corresponding to some points. So we construct more powerful constraint condition, the distance of three points, and the area of triangle of three points, further to select the corresponding point set. This constraint condition is independent of rigid transformation and will improve the reliability and efficiency of determining corresponding points, and its specific steps are as follows.

Assume there are three known reference points *m*_1_(*x*_1_, *y*_1_, *z*_1_), *m*_2_(*x*_2_, *y*_2_, *z*_2_), and *m*_3_(*x*_3_, *y*_3_, *z*_3_) and three corresponding points *n*_1_, *n*_2_, and *n*_3_ that need to be determined. Let *a*, *b*, and *c* represent the distance between *m*_1_, *m*_2_ or *m*_1_, *m*_3_ and *m*_2_, and *m*_3_, respectively. *S* is the area of triangle composed of *m*_1_, *m*_2_, *m*_3_. The computing function of the distance and the area are shown as follows:

(4) $$\begin{array}{c} a=\sqrt{{\left(x_{1}-x_{2}\right)}^{2}+{\left(y_{1}-y_{2}\right)}^{2}+{\left(z_{1}-z_{2}\right)}^{2}},\\ b=\sqrt{{\left(x_{1}-x_{3}\right)}^{2}+{\left(y_{1}-y_{3}\right)}^{2}+{\left(z_{1}-z_{3}\right)}^{2}},\\ c=\sqrt{{\left(x_{2}-x_{3}\right)}^{2}+{\left(y_{2}-y_{3}\right)}^{2}+{\left(z_{2}-z_{3}\right)}^{2}},\\ S=\sqrt{\frac{\alpha +b+c}{2}*\left(\frac{\alpha +b+c}{2}-b\right)*\left(\frac{\alpha +b+c}{2}-c\right)}. \end{array}$$

The distance constraint condition is:

(5) $$\begin{array}{c} \left|d_{m_{i}m_{j}}-d_{n_{i}n_{j}}\right|<\alpha ,\;\left(i,j=1,2,3\right), \end{array}$$

where *d*_*m*_*i*_*m*_*j*__ and *d*_*n*_*i*_*n*_*j*__ are the distances between any two points of the reference points *m*_1_, *m*_2_, and *m*_3_, and the corresponding points *n*_1_, *n*_2_, *n*_3_, respectively. *α* is a threshold defined. The area constraint condition is shown by:

(6) $$\begin{array}{c} \left|S\left(m\right)-S\left(n\right)\right|<\beta , \end{array}$$

where *S*(*m*) is the area of triangle composed of *m*_1_, *m*_2_, and *m*_3_; *S*(*n*) is the area of triangle composed of the three corresponding points *n*_1_, *n*_2_, and *n*_3_; and *β* is the set error.

Through the above stated constraint conditions, we can select the corresponding points *n*_1_, *n*_2_, and *n*_3_ of the reference points *m*_1_, *m*_2_, and *m*_3_.

#### 2.3.2. The Course Registration

Local coordinate system of one of the two image models is built by reference points in the reference model. Assume that *m*_1_ is the origin of coordinate and the direction from *m*_2_ to *m*_3_ is *X* axis direction. Local coordinates can be expressed by *T*_*m*_ = (*t*_1_(*m*), *t*_2_(*m*), *t*_3_(*m*)), where

(7) $$\begin{array}{c} t_{1}\left(m\right)=\frac{m_{3}-m_{1}}{\left|m_{3}-m_{1}\right|},\\ t_{2}\left(m\right)=\frac{\left(m_{3}-m_{1}\right)\times \left(m_{2}-m_{1}\right)}{\left|\left(m_{3}-m_{1}\right)\times \left(m_{2}-m_{1}\right)\right|},\\ t_{3}\left(m\right)=t_{1}\left(m\right)\times t_{2}\left(m\right). \end{array}$$

Similarly, the local coordinate built by corresponding points *n*_1_, *n*_2_, and *n*_3_ is *T*_*n*_ = (*t*_1_(*n*), *t*_2_(*n*), *t*_3_(*n*)). By formula deducing, we could obtain rotation matrix *R* and translation vector *T* corresponding to the transformation of coordinate *T*_*m*_ to *T*_*n*_:

(8) $$\begin{array}{c} R=T_{n}\left({T_{m}}^{T}\right), \end{array}$$

(9) $$\begin{array}{c} T=\frac{n_{1}+n_{2}+n_{3}}{3}-\frac{R\left(m_{1}+m_{2}+m_{3}\right)}{3}. \end{array}$$

Then, the matrices  *R* and *T* are applied to the two target point sets and we could realize the course registration.

### 2.4. The Precise Registration

The accuracy of coarse registration cannot satisfy the ultimate requirements [16, 17], so ICP algorithm is adopted for getting higher registration accuracy.

Assume that the feature point sets of Blue-light scanner dental model and CBCT dental model are *P* = {*p*_*i*_ | *p*_*i*_ ∈ *R* ^3^; *i* = 1,2, 3,…, *N*_*P*_} and *Q* = {*q*_*j*_ | *q*_*j*_ ∈ *R* ^3^; *j* = 1,2, 3,…, *N*_*Q*_}, respectively, where *N*_*P*_ and *N*_*Q*_ stand for the number of the elements (*N*_*P*_ ≤ *N*_*Q*_). *R*, *T* are the rotation matrix and translation vector, which are obtained through the coarse registration. Assume that *k* and *μ* stand for the iterate times and the precision threshold. Treat *P* as the target point set and *Q* as the reference point set. Then carry out the ICP algorithm to make precise registration that consists of the following stages.

**Step 1**

Given that *k* = 0, *P* ^0^ = *P*, *P* ^0^ = {*p*_*i*_^0^ | *p*_*i*_^0^ ∈ *R* ^3^; *i* = 1,2, 3,…, *N*_*P*_}, *R* ^0^ = *R*, and *T* ^0^ = *T*, use *k*-*d* tree method to calculate the corresponding point set *D* ^0^ = {*q*_*j*_^0^ | *j* = *i* = 1,2, 3,…, *N*_*P*_}, which belongs to *Q*, and each point *q*_*j*_^0^ is with the least Euclidean distance to the point *p*_*i*_^0^  (*i* = 1,2, 3,…, *N*_*P*_). All the corresponding points must be unique. Define the initial residual error; that is, *r* ^0^ = (1/*N*_*P*_)∑_*i*=1_^*N*_*P*_^||*p*_*i*_^0^ − *D* ^0^(*i*)||.

**Step 2**

Use *R* ^*k*^ and *T* ^*k*^ transform point set *P* ^*k*^ to obtain the point set *P* ^*k*+1^, where *P* ^*k*+1^ = *R* ^*k*^ *P* ^*k*^ + *T* ^*k*^. Then calculate the corresponding point set *D* ^*k*+1^ = {*q*_*j*_^*k*+1^ | *j* = *i* = 1,2, 3,…, *N*_*P*_}, which also belongs to *Q*, and each point *q*_*j*_^*k*+1^ is with the least Euclidean distance to the point *p*_*i*_^*k*+1^. Then compute the residual error *r* ^*k*+1^ = (1/*N*_*P*_)∑_*i*=1_^*N*_*P*_^||*p*_*i*_^*k*+1^ − *D* ^*k*+1^(*i*)||.

**Step 3**

Judge whether the precision meets the requirements after iterating *k*(*k* ≥ 0) times. When |*r* ^*k*+1^ − *r* ^*k*^ | ≤*μ*, is satisfied stop iterating. Otherwise, continue to Step 4.

**Step 4**

Calculate the rotation matrix *R* ^*K*+1^ and translation vector *T* ^*K*+1^ of transform relation between *P* ^*k*+1^ and *D* ^*k*+1^ through the Singular Value Decomposition (SVD) method [18]. Then renew *k* = *k* + 1, and transfer to Step 2.

The Singular Value Decomposition (SVD) method, used to calculate the rotation matrix and translation vector, is demonstrated in the appendix.

**Figure 2:**
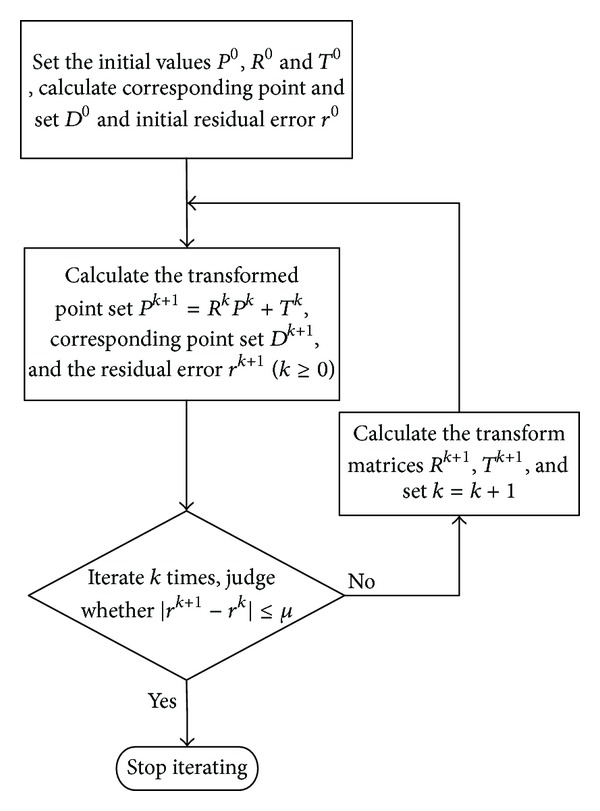
The flow chart of precise registration.

Figure 2 is the flow chart of precise registration.

## 3. The Registration of CBCT Dental Model and inEos Blue Dental Model

The experimental procedures include acquiring data, preprocessing, extracting feature points, and registration.

### 3.1. Data Acquisition and Preprocessing

**Figure 3:**
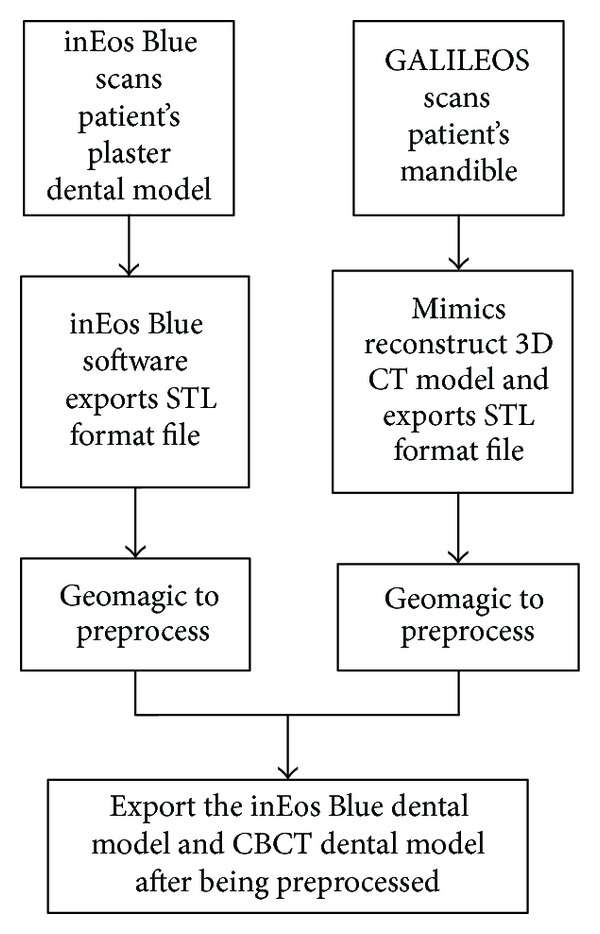
The flow chart of data acquisition and preprocess.

**Figure 4:**
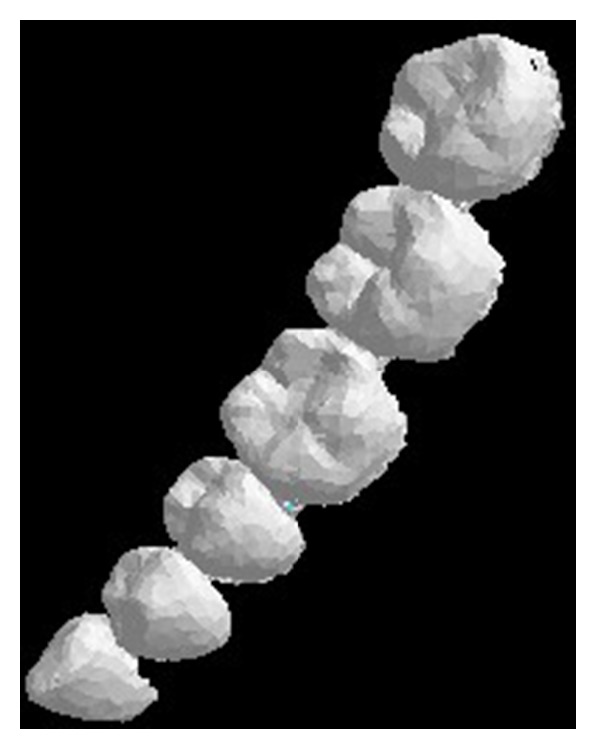
inEos Blue dental model.

The main procedures of data acquisition and preprocessing are shown in Figure 3. Firstly, using 3D Sirona inEos Blue Blu-ray scanner (resolution is 0.02 mm) patient's plaster dental model is scanned. Next, the own software of scanner is applied to obtain STL format dental file. Geomagic software is used to preprocess the exported file, and then, export the preprocessed file, which is STL that format includes dental crown only. The result is shown in Figure 4.

**Figure 5:**
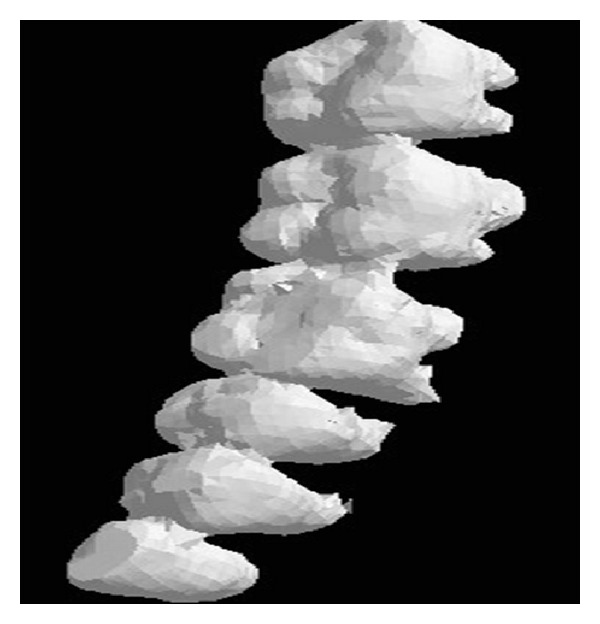
CBCT dental model.

Sirona GALILEOS system (resolution is 0.1 mm) is adopted to scan the same patient's mandible to obtain the CT scanning data. The data is DICOM format and then it is imported into commercial processing software called Mimics, which is used to reconstruct 3D CT model. After preprocessing, the 3D mandible model with STL format is exported.  Figure 5 shows the CBCT dental model with both dental crown and root.

### 3.2. Extracting the Feature Points

**Figure 6:**
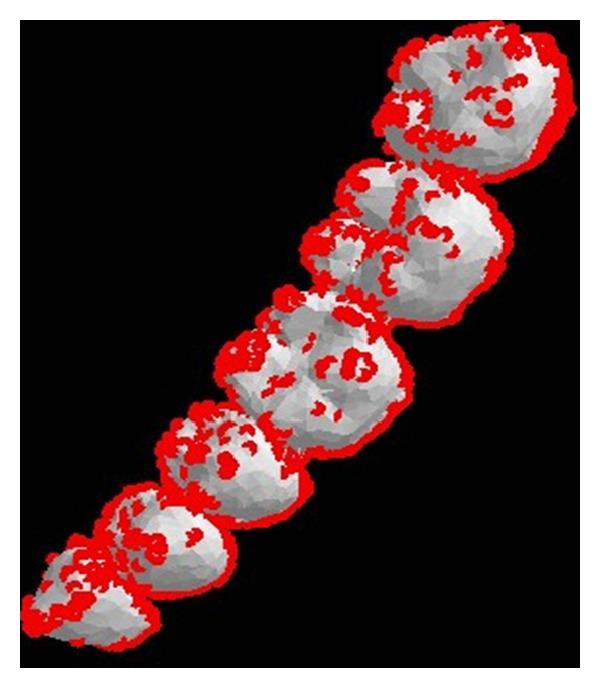
The extracted feature point set *P*.

The inEos Blue dental model, showed in Figure 4 as the sample, set the threshold *α* = 0.01. Calculate the vertex curvature of the dental crown using the improved Voronoi method mentioned before. When the curvature value of any point is more than *α*, mark it as a feature point and put it into point set list, which is denoted by *P*. The red dots showed in Figure 6 represent the feature points.

### 3.3. Coarse and Precise Registration

**Figure 7:**
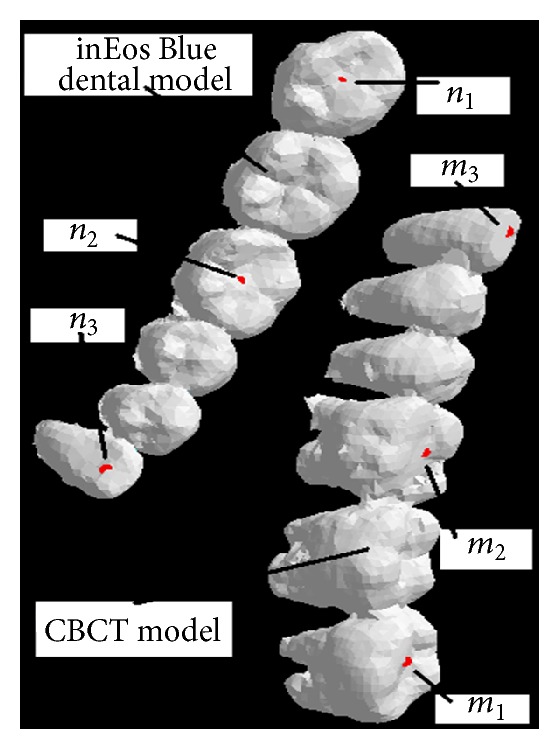
The initial position of model and corresponding points.

The resolution of 3D inEos Blue dental data is higher than CBCT data, while CBCT model includes the integral dental data. The improved three point's translational transformation method is used to realize coarse registration, and the results are shown in Figure 7. Select three corresponding points *m*_1_, *m*_2_, and *m*_3_ as reference points from CBCT dental model and set the threshold 0.00015. Then extract the corresponding points *n*_1_, *n*_2_, and *n*_3_ and calculate rotation matrix *R* and translation vector *T*. The results are listed in Table 1.

**Figure 8:**
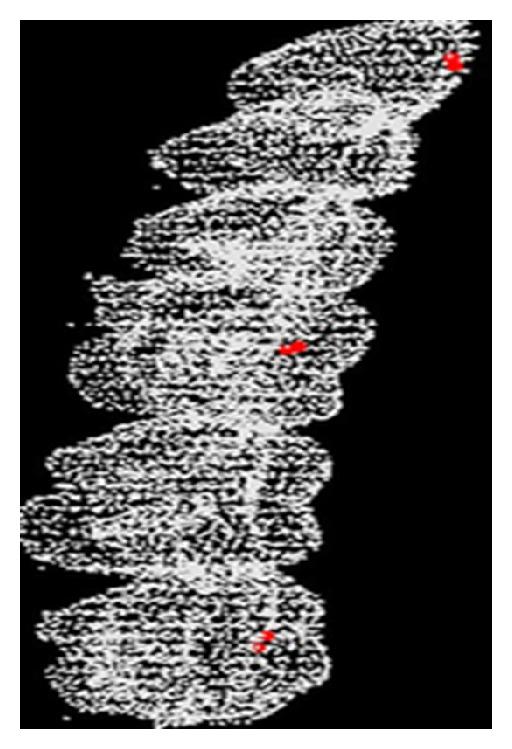
The results of coarse registration.

Formula (8) is used to realize coarse registration, and Figure 8 is the result of coarse registration.

**Figure 9:**
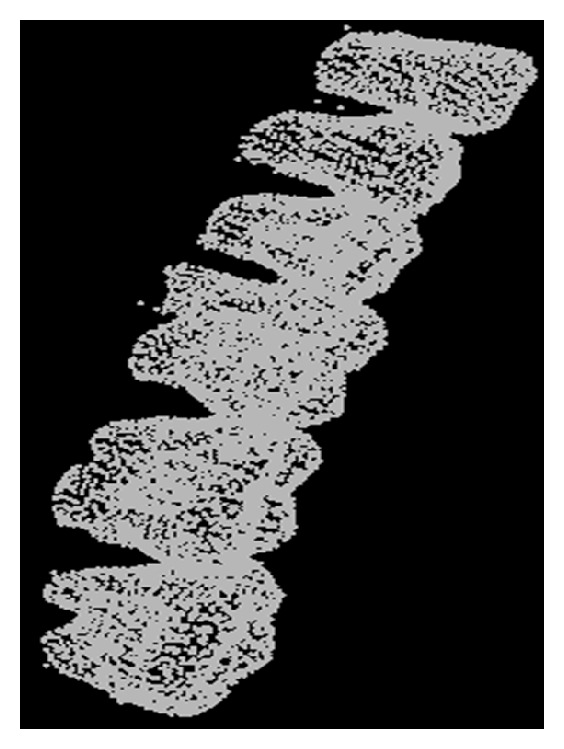
The result of ICP precise registration.

Based on the results of coarse registration, the precise registration is carried out for improving the accuracy of registration further.  Figure 9 is the result, which reveals that the two models can be fused more accurately.

## 4. Discussions

Supported by the VC6.0 platform, we used VC++ and OpenGL to conduct simulation and experiments.

### 4.1. The Accuracy of Registration

We show dental models registration results to evaluate the accuracy of our algorithm, and the average distance of corresponding points is calculated. For the first experiment the average distance of the coarse registration, shown in Table 2, is 0.7291 mm while it is reduced to 0.3835 mm after precise registration.

Other four groups of patient's dental image data are carried out by the same procedure, which also conclude inEos Blue scanner and CBCT 3D dental models and yield the same conclusion. The total average distance of the precise registration is 0.8266 mm, compared with 1.4486 mm of the coarse registration. Comparing with the coarse registration accuracy, the precise one is improved significantly, which basically meets the requirements of helping dentist for designing remedies.

### 4.2. The Reliability and Advantages of the Feature Extraction

Considering the characteristic of the STL model data itself, we take the CBCT 3D dental model as the reference model and adopt the constraint-based curvature method. As mentioned earlier, extract the corresponding feature points automatically in the inEos Blue scanner dental model. Experimental results show that the method could extract the corresponding feature points correctly and be operated simply and reliably. Moreover, it efficiently overcomes the shortages of both the traditional surface-based marking method and the corresponding manually selected feature points method.

### 4.3. Further Extension and Improvement

Multimodality image registration and fusion technology can make full use of the complementary characteristics which are provided by different imaging models. The technology could perfectly integrate the image data to provide abundant information for clinical diagnosis. The improved ICP method proposed in this paper realized the feature points extraction and image registration successfully between the inEos Blue scanner dental model and the CBCT 3D dental model. Besides, it provides reliable registration method in the computer-aided dental implant design system. The research and the method in this paper will be the reference and application for similar studies and other multimodality image registrations.

Imperfectly, in the process of extracting feature points with curvature characteristics, the threshold has to be appropriately adjusted according to the model complexity and the accuracy requirements, which make the registration accuracy to a larger degree depend on the reasonable choice. The adaptive threshold selection method demands our further research.

## 5. Conclusion

As the traditional ICP algorithm cannot realize the image registration with different resolution, we propose an improved ICP algorithm in this paper. The procedure of this algorithm used in the registration of 3D CBCT dental model and 3D inEos Blue scanning dental model is discussed in detail. The experimental results verified that this method can guarantee a high accuracy in registration.

## Figures and Tables

**Table 1:** Rotation matrix *R* and translation vector *T*.

Variable name	Variable value
Rotation matrix *R*	$\left(\begin{array}{ccc} -0.82982 & 0.24221 & -0.50271\\ -0.33881 & -0.93451 & 0.10903\\ -0.44338 & 0.26080 & 0.85755 \end{array}\right)$
Translation vector *T*	$\left(\begin{array}{ccc} 124.9116 & -65.0124 & 63.4470 \end{array}\right)$

**Table 2:** Compared results on the coarse registration and precise registration.

Corresponding point pairs	Average distance after coarse registration (mm)	Average distance after precise registration (mm)
Pair-1	0.5832	0.4255
Pair-2	0.6358	0.5462
Pair-3	0.6975	0.4344
Pair-4	0.1928	0.0659
Pair-5	0.4673	0.2843
Pair-6	0.6750	0.4363
Pair-7	0.7213	0.4947
Pair-8	0.5918	0.3709
Pair-9	0.5686	0.3144
Pair-10	0.6847	0.5076
⋮	⋮	⋮
Pair-1308	0.5967	0.4632

Average distance (mm)	0.7291	0.3835

## Appendix

Assume the unit quaternion is *a* = *a*_0_ + *a*_1_ *i* + *a*_2_ *j* + *a*_3_ *k*. The quaternion expression after coordinate system unit vector (*v*_1_, *v*_2_, *v*_3_) rotated angle *θ* is

(A.1) a=cos(θ2)+v1sin(θ2)i+v2sin(θ2)j+v3sin(θ2)k.

The corresponding rotation matrix is

(A.2) R(a)=(a02+a12−a22−a322(a1a2−a0a3)2(a1a3+a0a2)2(a1a2+a0a3)a02+a22−a12−a322(a2a3+a0a1)2(a1a3−a0a2)2(a2a3+a0a1)a02+a32−a12−a22).

Assume the centroids of two point sets *P* ^*k*+1^ and *D* ^*k*+1^ are *C*_*P*_ = (1/*N*_*P*_)∑_*i*=1_^*N*_*P*_^ *p*_*i*_^*k*+1^ and *C*_*D*_ = (1/*N*_*P*_)∑_*j*=1_^*N*_*P*_^ *q*_*j*_^*k*+1^, respectively. The covariance matrix between the point set *P* ^*k*+1^ and *D* ^*k*+1^ is = (1/*N*_*P*_)∑_*i*=1_^*N*_*P*_^(*p*_*i*_^*k*+1^ − *C*_*P*_)(*q*_*i*_^*k*+1^ − *C*_*D*_), and trace(*C*) represents the trace of matrix *C*. Define symmetric matrix *H*:

(A.3) $$\begin{array}{c} H=\left(\begin{array}{cccc} \text{trace}\left(C\right) & c_{12}-c_{21} & c_{20}-c_{02} & c_{01}-c_{10}\\ c_{12}-c_{21} & 2c_{00}-\text{trace}\left(C\right) & c_{01}+c_{10} & c_{02}-c_{20}\\ c_{20}-c_{02} & c_{01}+c_{10} & 2c_{11}-\text{trace}\left(C\right) & c_{12}+c_{21}\\ c_{01}-c_{10} & c_{02}+c_{20} & c_{12}+c_{21} & 2c_{22}-\text{trace}\left(C\right) \end{array}\right). \end{array}$$

Using Jacobi method, calculate the eigenvalue and the eigenvector of matrix *H*. The corresponding eigenvector of the maximum eigenvalue is the quaternion $a={\left(\begin{array}{cccc} a_{0} & a_{1} & a_{2} & a_{3} \end{array}\right)}^{T}$, which meets the requirement. As these are known, then we can calculate rotation matrix *R*(*a*), that is, *R* ^*k*+1^ = *R*(*a*); and the translation vector is *T* ^*k*+1^ = *C*_*D*_ − *R* ^*k*+1^ *C*_*P*_.
